# Harnessing prion-inspired amyloid self-assembly for sustainable and biocompatible proton conductivity†

**DOI:** 10.1039/d4na00303a

**Published:** 2024-04-17

**Authors:** Susanna Navarro, Andreu Andrio, Marta Diaz-Caballero, Salvador Ventura, Vicente Compañ

**Affiliations:** a Institut de Biotecnologia i Biomedicina and Departament de Bioquímica i Biología Molecular, Universitat Autónoma de Barcelona 08193 Bellaterra Barcelona Spain Susanna.Navarro.Cantero@uab.es; b Dpto. Física. Universitat Jaume I Avda. Sos, Baynat s/n Castellon 12071 Spain; c Escuela Técnica Superior de Ingenieros Industriales, Departamento de Termodinámica Aplicada, Universitat Politècnica de València Camino de Vera s/n 46020 Valencia Spain vicommo@ter.upv.es

## Abstract

Protein-based materials have emerged as promising candidates for proton-conducting biomaterials. Therefore, drawing inspiration from the amino acid composition of prion-like domains, we designed short self-assembling peptides incorporating the (X-Tyr) motif, with X representing Asn, Gly and Ser, which form fibrillar structures capable of conducting protons. In this study, we conducted an analysis of the conductivity capacity of these fibers, with a focus on temperature and frequency dependence of conductivity. The loss tangent curves data and the electrode polarization model with the Debye approximation were employed to calculate transport properties, including conductivity, diffusivity, and density of charge carriers. Results revealed the prion-like fibers can transport protons more efficiently than biomaterials and other synthetic proton conducting materials, and that a significant increase in conductivity is observed with fibrillar orientations. The temperature dependence of conductivity of the peptides, measured in wet conditions, showed conductivities following the trend *σ*(NY7) < *σ*(GY7) < *σ*(SY7), in all the range of temperatures studied. The Arrhenius behavior, and the activation energy associated with conductivity followed the trend: *E*_act_ (SY7) = 8.2 ± 0.6 kJ mol^−1^ < *E*_act_ (GY7) < 13 ± 5 kJ mol^−1^ < *E*_act_ (NY7) = 31 ± 7 kJ mol^−1^, in different range of temperatures depending of the peptide. Furthermore, the diffusion coefficient correlated with increasing temperature in GY7 and SY7 fibers for temperatures compress between 20 °C and 80 °C, while NY7 only below 60 °C. However, it is noteworthy that the diffusivity observed in the SY7 peptide is lower, compared to GY7 and NY7 presumably due to its enlarged length. This observation can be attributed to two factors: firstly, the higher conductivity values observed in SY7 compared to GY7 and NY7, and secondly, to the value of relation 
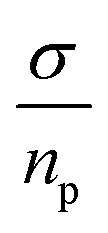
 observed of cations present in the peptide SY7 compared with GY7 and NY7, which in turn is dependent on temperature. In light of these findings, we envision our prion-inspired nanofibers as highly efficient proton-conducting natural biopolymers that are both biocompatible and biodegradable. These properties provide the opportunity for the development of next-generation bioelectrical interfaces and protonic devices.

## Abstract 2

Protein-based materials have emerged as promising candidates for proton-conducting biomaterials.

## Graphical abstract

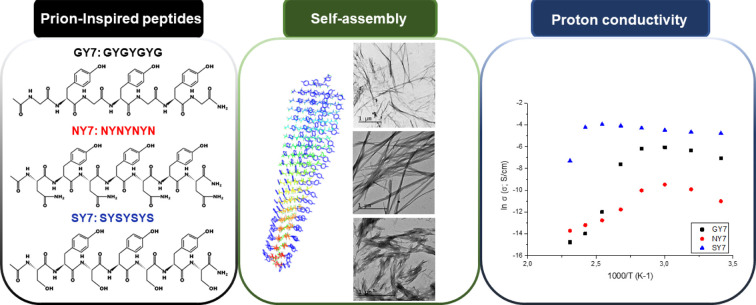

## 1. Introduction

Proton conduction is a widely studied phenomenon that is essential for biological processes in living organism including the production of adenosine triphosphate during mitochondrial respiration, transmission of signals in the nervous system and photosystems II, among others. What is common in all those processes are proteins. Peptides and proteins are the battle-horses for a wide range of cellular functions, encompassing molecular recognition, catalysis, replication, mechanical responsiveness, enzymatic activity, transmembrane channel transport, and signal transduction. Therefore, their multifaceted roles make them essential components in the cell.

Among them, amyloid proteins have been traditionally linked to diseases, including neurodegenerative disorders and systemic amyloidosis.^1^ A particular class comprises prions, which are a specific subset of amyloids. They share a supramolecular fibrillar structure characterized by a cross-β fold and stabilized by a dense network of hydrogen bonds, exhibiting unique self-templating and propagating properties.^1^ The existence of prion domains (PrDs), displaying low composition complexity and enriched in polar and uncharged amino acids, plays a key role in the amyloid formation observed in prions and prion-like proteins.^2^ Thus, we have previously described that short sequence stretches of moderate amyloid propensity within PrDs are necessary and sufficient to drive the autonomous self-assembly of these domains.^3^

In recent decades, there has been a growing interest in the development of novel functional structures derived from peptides and their derivatives. This interest revolves around two key approaches: one involves replicating the functions of peptides and proteins, while the other entails devising entirely new design principles.

Accordingly, we designed a new class of short peptides featuring a (X-Tyr) motif, where X can be Asn, Gly and Ser, that is frequently observed in amyloidogenic PrDs.^4^ The binary patterned heptapeptides (Ac-NYNYNYN-NH_2_, Ac-SYSYSYS-NH_2,_ and Ac-GYGYGYG-NH_2_), from now on referred as NY7, SY7 and GY7 respectively, recapitulate PrDs self-assembly properties under mild experimental conditions, including neutral pH, room temperature, and ambient pressure. In addition, their ordered high aspect ratio spatial arrangement makes them promising building blocks for the creation of biomaterials with properties that include high stability, strength, biocompatibility, cost-effectiveness, and environmental friendliness.^4^

The self-assembly of peptides containing tyrosine (Tyr) residues results in fibrillar structures displaying catalytic properties, such as pyrrole polymerization^4^ and proton transport activities.^5^ Despite not being traditionally considered protonable, the incorporation of tyrosine into scaffolds can enhance proton transport through electron-coupled proton transfer (ECPT). This is attributed to tyrosine's capacity to both donate and accept electrons, leading to the formation of stable radicals that facilitate proton transfer *via* simultaneous electron exchange. Among the amino acids, tyrosine stands out as the primary redox-active amino acid, thanks to its redox-active phenol group, which has been suggested to play a significant role in numerous enzymes.

In this context, early investigation into proton conducting bioelectronics have previously reported the conductivity of both globular and fibrous proteins^6,7^ with water content being identified as a crucial contributor to their conductivity in silk and collagen.^8^ Additionally, mimics of eumelanins, made of Tyr, exhibit redox activity and can undergo oxidation and polymerization, resulting in substantial proton conductivity and hydration-dependent electrical current.^9^ Thus, the hydrogen bonding of tyrosine residues with surrounding residues is key to proton transfer in many biological systems, and the degree of overlap between donor and acceptor proton vibrational wavefunctions is greatly influenced by the distance over which proton transfer occurs.^10^

With the aim of increasing the conductivity of polymeric membranes our group has been a pioneer in the development of nanocomposite membranes that incorporate surface functionalized nanofibers of polyvinyl alcohol (PVA) on Nafion.^11^ These nanofibers were chemically modified with 4-formyl-1,3-benzene disulfonic for the incorporation of acid groups enhance the interaction between the nanofibers and the polymeric matrix through hydrogen bonding by sulfonic acid moieties. The obtained results revealed the significant influence of the water used as solvent on the crosslinking temperature within the nanocomposite obtained.^11,12^

In the present work, we aim to establish a dependable method for determining the protonic conductivity, the concentration of mobile charge carriers and protonic diffusivity in prion-inspired fibers. We achieve this by leveraging impedance and dielectric spectroscopic data, a technique previously employed to characterize protonic conductivity in polymeric membranes. Our approach involves analyzing the space charge relaxation of the self-assembled peptides positioned between two blocking electrodes, and employing a modelling framework. This methodology builds upon the models proposed by Schütt^13,14^ and Coelho^15^ for ion-conducting glasses, which have been further generalized by Jönsson *et al.*^16^

In previous studies, broadband dielectric spectroscopy has been employed to investigate the relationship between diffusivity, charge carrier density, and Debye length in relation to the structural dynamics of supported ionic liquid-like phases (SILLPs). In this investigations, the influence of electrode polarization (EP) has been taken into account using a single Debye relaxation model, as demonstrated in the analysis conducted using Trukhan theory trough the work published by Compañ *et al.*,^17^ Sorensen *et al.*,^18^ and MacDonald,.^14,19–25^ By incorporating the EP approach and fitting the peaks corresponding to the maximum of tan *δ*, we were able to determine the diffusivity, charge carrier density and Debye length. These parameters provided insights into the cumulative process occurring in the system, resulting from interactions between charge carriers and the mobile ion concentration in the peptides. Additionally, we investigated the transport properties (*i.e.*, protonic conductivity) using dielectric spectroscopy to explore whether the conductivity of the peptides could be related with a higher ratio of stable inter-sheet hydrogen bonds between side chain-backbone and side chain–side chain interactions.

Considering that higher degrees of crosslinking are expected to alter the interaction between the fibers and the matrix, potentially impeding proton conduction, the opposite effect occurs with lower levels of crosslinking in the peptides. Lower crosslinking allows for the simultaneous presence of a higher concentration of water molecules on the surface of the Tyr-rich fibers, which, in turn, facilitates the proton transport mechanism.

This phenomenon can be likened to what is observed in relaxed muscle fibers, where water molecules are tightly and orderly bound to the protein, similar to ice. However, when the muscle contracts, these bound water molecules are released and become “free.” In its contracted state, a muscle fiber can sustain transport processes through the Grotthuss mechanism within the hydrated sheath of its protein molecules. In this state, electrical impulses can be transmitted through proton conduction and the exchange of electrons, which can be generated through hydrogen bond reactions with water molecules, all facilitated by the Grotthuss mechanism.^26,27^ It can be stated that the binding and release of water molecules act as a mechanism to control the transmission of energy, with the energy required decreasing as the number of water molecules increases.

Based on our findings, it is concluded that the linearity of Tyr-rich fibers and the water content are pivotal factors influencing conductivity levels, resulting in either decreased or increased values. Within this framework, we introduce prion-inspired amyloid-like nanostructures possessing ECPT capabilities, holding promise for a wide array of potential applications in energy conversion, catalysis, sensing, and the biomedical field. Specifically, in the field of biomedical applications, these biocompatible organic nanomaterials could prove invaluable in drug delivery systems, biosensors, cell tissue engineering, and bioelectrical interfaces.

## 2. Materials and methods

### 2.1. Preparation of amyloid fibers

The synthetic heptapeptides NY7, SY7 and GY7 (Ac-NYNYNYN-NH_2_, Ac-SYSYSYS-NH_2,_ and Ac-GYGYGYG-NH_2,_ respectively) with >99% purity (RoyoBiotech) were acetylated at the N-terminal, and amidated at the C-terminal to neutralize terminal charges. Lyophilized peptides were resuspended in 1,1,1,3,3,3-hexafluoropropanol at a final concentration of 10 mM, and further dissolved in 100 mM KH_2_PO_4_, pH 7.0 at a final concentration 250 μM for NY7 and SY7 and at 500 μM for GY7 peptides. Aggregated peptides were obtained upon 7 days incubation at RT, and soluble monomer species were removed by centrifugation.

### 2.2. Amyloid dyes binding

For Th-T binding assay, incubated peptides were diluted 1 : 10 in KH_2_PO_4_, pH 7.0 buffer, and Thioflavin-T (Th-T) was added to a final concentration of 25 μM. Th-T emission fluorescence was detected in the range 460–600 nm on a Jasco FP-8200 fluorescence spectrophotometer (Jasco Corporation, Japan), using an excitation wavelength of 445 nm and with an excitation and emission bandwidth of 5 nm.

The Congo Red (CR) absorbance spectra were obtained by mixing 20 μM CR with a final concentration of fibers of 20 μM. The absorbance spectra were recorded in a SPECORD 200 Plus spectrophotometer (Analitik Jena) at the range from 375 to 700 nm. The spectrum of buffer was acquired as the signal baseline and proteins alone were used to subtract the scattering contribution.

### 2.3. Transmission electron microscopy (TEM)

For preparing samples for TEM analysis, 10 μL of the aged fibril solution was placed on a carbon-coated copper grid for 10 minutes and the excess liquid was wiped away using filter paper. Then, grids were negatively stained with addition of 10 μL of a 2% w/v uranyl acetate solution on top for 1 min, and excess stain was removed by touching the torn edge of a paper strip to the edge of the grid. In heated samples, fibers were warm up to 110 °C for 10 min, deposited on carbon-coated copper grids and negatively stained as previously described. Samples were thoroughly examined using a JEM 1400 transmission electron microscope (made by JEOL Ltd in Japan) with an operating voltage of 120 kV and the images were captured using a CCD GATAN ES1000W Erlangshen camera (manufactured by Gatan Inc., in the United States).

### 2.4. Fourier transformed infrared spectroscopy

The prepared fibers were subjected to high-speed centrifugation at 12.000*g* for 30 minutes and then resuspended in water. The samples were applied onto an ATR crystal and allowed to dry. The FTIR experiments were performed using a Bruker Tensor 27 FTIR instrument and a Specac Golden Gate MKII ATR accessory. Each IR spectrum was obtained by acquiring 32 scans at a resolution of 1 cm^−1^. Data analysis was performed using the OPUS MIR Tensor 27 software, and the spectra were fitted using a nonlinear peak-fitting equation and the Peak Fit package v4.12. The amide I region (1700 to 1600 cm^−1^) was analyzed using the second derivative deconvolution method in PeakFit package v4.12 to calculate the area of each Gaussian curve.

### 2.5. Conductivity measurements

#### 2.5.1. Sample preparation

In this study, we conducted measurements on two distinct sample categories: dry ones and wet ones. The dry samples were obtained by compacting the initial raw powder into thin discs measuring 12 mm in diameter and approximately 0.7 mm of height. The preparation of wet samples, on the other hand, commenced with the regrinding of the raw material to achieve an exceptionally fine powder consistency. Subsequently, 100 mg of this finely ground powder was meticulously blended with 40 mg of ultrapure water, resulting in the formation of a homogenous wet paste. It is noteworthy that the visual appearance of the wet paste remained consistent throughout the entire measurement process.
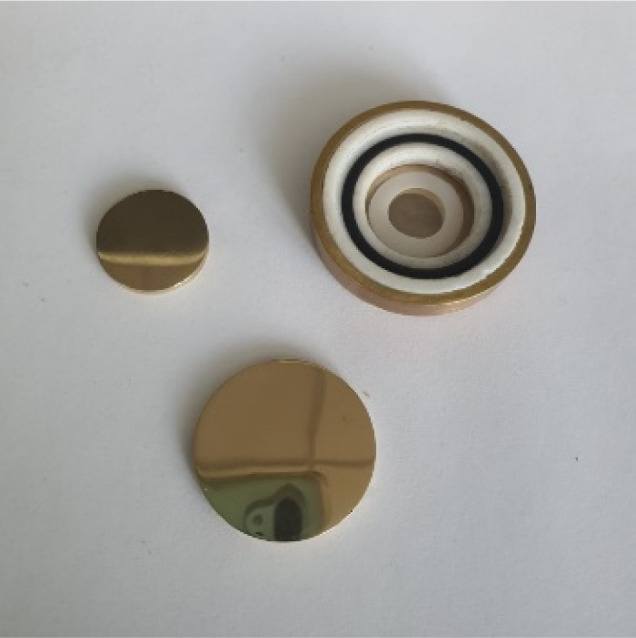


In the photograph, is showed the cell which after contain the sample is insert in the electrode apparatus is depicted. This device facilitates optimal contact between the two faces of the samples and two flat metallic surfaces. Furthermore, it establishes a hermetic chamber to prevent desiccation of the wet samples during measurements. In the case of wet samples, a Teflon spacer was employed to set up both, sample thickness and contact areas between the electrodes and sample. So, the paste adopts the shape of a very thin cylinder.

#### 2.5.2. Dielectric spectroscopy measurement

The dielectric study of the samples prepared, was conducted to obtain the conductivity, in dry and wet conditions in the transversal direction. For this, the measurements were carried out using a broadband dielectric spectrometer (Concept 80, Novocontrol Technologies, Hundsangen, Germany) integrated with an SR 830 lock-in amplifier with an Alpha dielectric interface analyzer. The analyzer was supported by Quatro temperature controller using nitrogen as heating agent and providing a temperature stability around 0.1 K. The conductivity measurements of the samples were performed in the temperature range compress between 20 and 160 °C in steps of 20 °C by electrochemical impedance spectroscopy (EIS). The frequency interval considered was 10 MHz to 0.1 Hz, applying an electric field in the range between 4 and 10 V cm^−1^ for all the samples analyzed. The membranes thickness was measured afterwards using a digital micrometer, taking the average of five measurements at different parts of the sample surface. Then, the samples were placed between two gold electrodes coupled to the spectrometer.

Measurements were made on dry and wet samples. Dry samples have been measured several times and the same conductivity values can be obtained any time. On the other hand, in order to achieve good reproducibility with wet samples, the amount of water must be kept at a suitable level. This is not very problematic below 100 °C, if the measurements are sufficiently fast, but becomes considerably more difficult as the temperature rises. To make the measurements with the wet samples, an airtight cell was used to minimise drying out of the samples, such as has been described previously. Several cycles of temperature sweep between 20 and 100 °C were performed to check the reproducibility at these temperatures. The 100 °C limit in these tests was set to ensure that the same sample could withstand the cycles without damage. Measurements between 20 °C and 160 °C are revealing in terms of the conductive behaviour of the sample but require the sample to be discarded after the measurement, preventing measurements of several cycles up to that temperature. Supplementary Fig. SI1 and SI2† shows the variations of the real part of the conductivity at various frequencies for the samples NY7 and GY7, respectively, in one of the cycling measurements. The results observed have shown that the experimental procedure followed for the determination of the conductivities of the fibers has been satisfactory, and in all cases, the measurements were reproducible with an uncertainty lower than 5%.

## 3. Results

### 3.1 Physicochemical characterization of prion-inspired amyloid self-assemblies

**Fig. 1:**
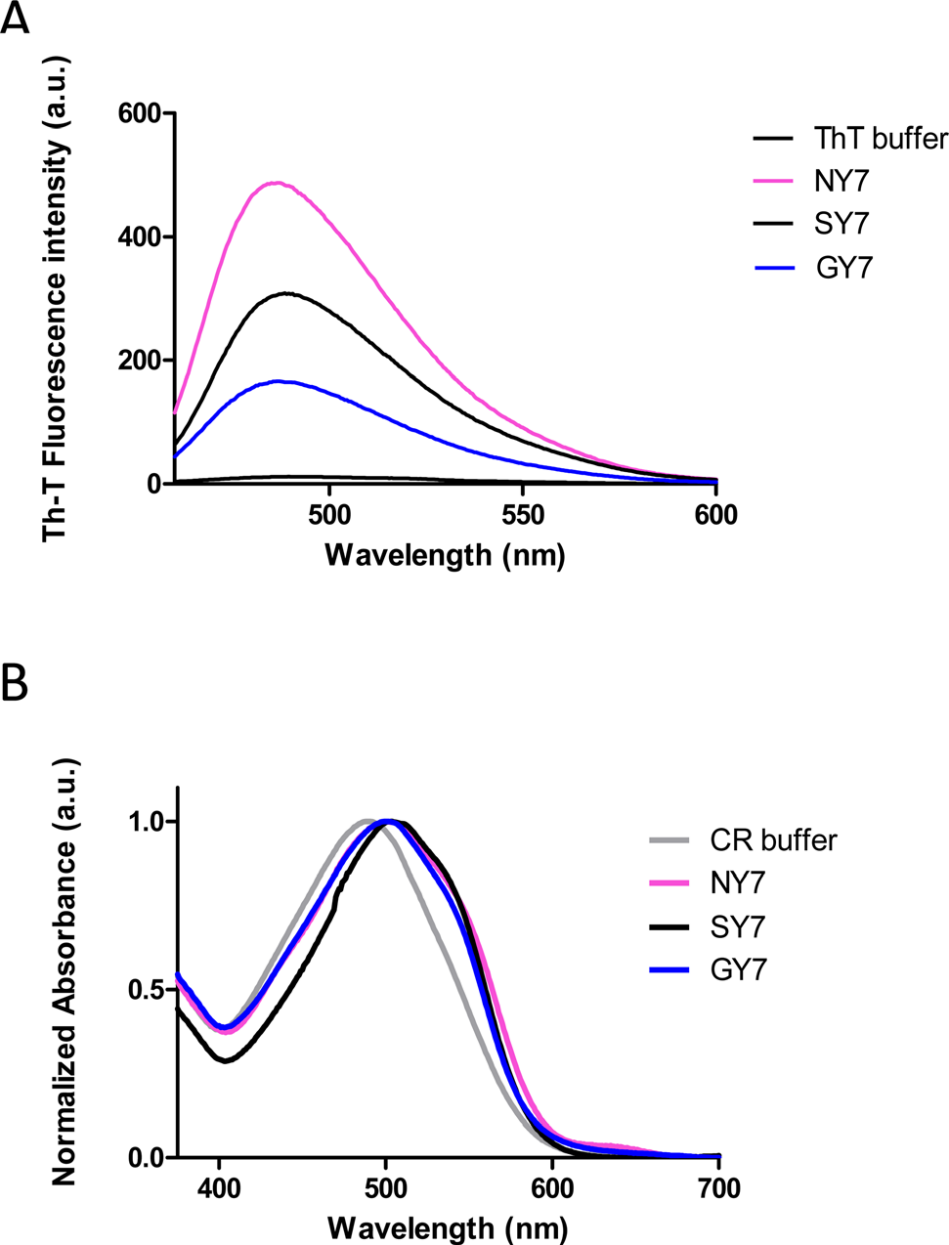
Biophysical characterization of Tyr-enriched self-assembled heptapeptides (NY7, SY7, and GY7). (A) Fluorescence emission spectra of Th-T recorded upon 445 nm excitation. (B) Normalized absorbance spectra of CR.

The generation of amyloid fibers using the NY7, SY7, and GY7 synthetic peptides was assessed using Thioflavin-T (Th-T) and Congo Red (CR) dyes. Th-T can specifically interact with β-sheet structures in amyloid fibrils and fluoresce upon binding. The intensity of Th-T fluorescence reports on the amount and arrangement of amyloid fibrils present in the sample. As it is shown in Fig. 1A, all incubated peptides demonstrated a significant increase in Th-T fluorescence emission intensity, with minor variations attributed to the amino acid sequence. To confirm the presence of amyloid assemblies, we also utilized the CR dye. The addition of CR to the incubated samples resulted in a red-shift of the absorption spectra (Fig. 1B). These data converge to demonstrate the formation of amyloid-like structures by NY7, SY7, and GY7 peptides.

**Fig. 2:**
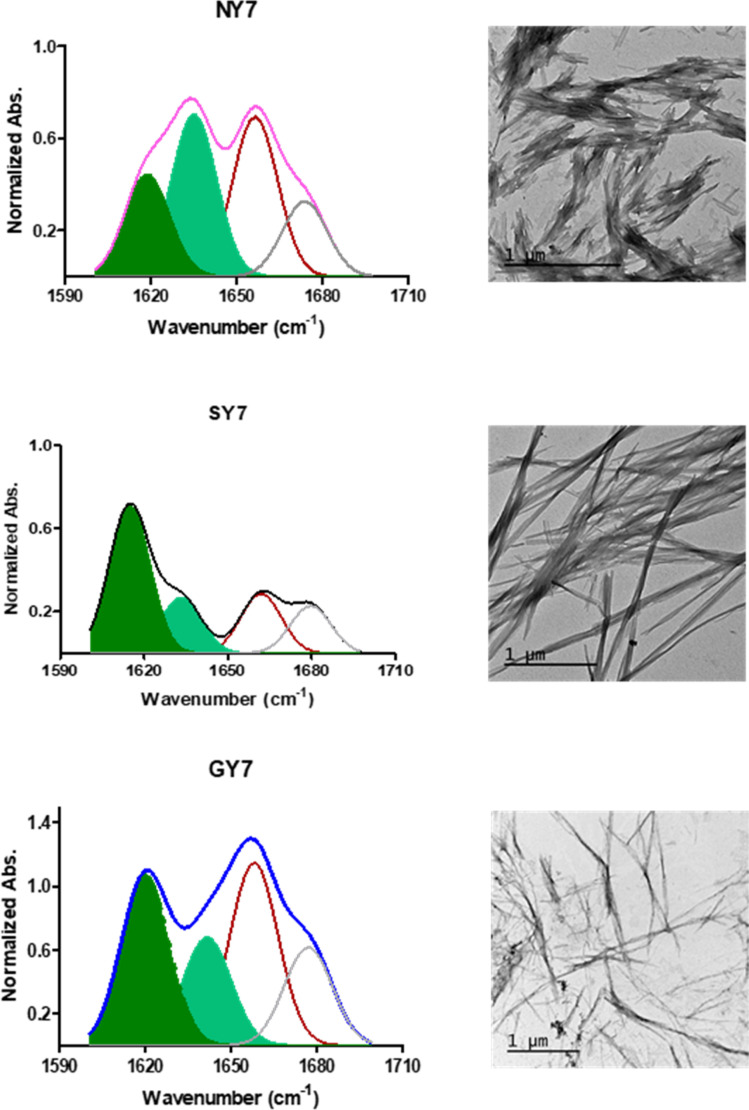
Conformation and morphology of Tyr-enriched heptapeptides fibers (NY7, SY7, and GY7). (Left panel) FT-IR absorbance spectra in the amide I region NY7 (pink), SY7 (black), and GY7 (blue) respectively. The secondary structure of fibers was derived from the deconvolution of the second-derivative and the fitted individual bands are shown. Bands corresponding to intermolecular β-sheet conformation are coloured in green. (Right panel) representative TEM micrographs. Scale bar 1 μm.

Subsequently, we corroborated the β-sheet enriched conformation in the assemblies by Attenuated Reflectance Fourier-Transform Infrared (ATR-FTIR) spectroscopy. Specifically, we recorded the amide I region of the spectrum (1700–1600 cm^−1^), which corresponds to the absorption region of the carbonyl peptide bond group of the protein backbone (Fig. 2).

Deconvolution of the spectra allowed us to assign the relative contribution of the different secondary structure elements. As shown in Table 1, all incubated heptapeptides displayed signals indicative of the formation of β-sheet structure, coming both from the intermolecular β-sheet region in the range 1615–1636 cm^−1^ and the β-turn region comprised between 1675 and 1682 cm^−1^, contributing to >50% of the spectral area in all the cases.^28^ Interestingly, no band indicative of antiparallel β-sheet was detected (∼1685–1690 cm^−1^) in any of the samples, thus indicating that the detected β-strands in the self-assembled peptides might adopt preferentially a parallel disposition.

**Table 1:** Secondary-structure assignments of Amide I band components by ATR-FTIR spectroscopy

Band assignment	NY7 (% area)	SY7 (% area)	GY7 (% area)
β-sheet (1623–1641 cm^−1^)	48	63	49
β-sheet (1674–1680 cm^−1^)	14	18	32
Disordered/α-helix (1642–1657 cm^−1^)	30	15	17

Contribution of the secondary structure elements from the deconvoluted absorbance spectra shown in Fig. 2.

Peptides can self-assemble into distinct dispositions, resulting in a range of morphologies. Thus, negative-staining and transmission electron microscopy (TEM) visualization was used to assess the macromolecular architecture of the peptide assemblies (Fig. 2). In agreement with the secondary structure analysis, all peptides showed a long, thin and unbranched fibrillar morphology. Nevertheless, it was observed that NY7 peptides assembled laterally, in a sort of fiber-bundle disposition. SY7 fibrils are longer than NY7 ones, a feature that according to our previous computational studies^29^ may respond to the higher structural flexibility of SY7 fibrils, which would decrease their brittleness compared to the more rigid NY7 fibrils. On the other hand, GY7 fibrils appear thinner and less electron-dense than those of NY7 and SY7 peptides, consistent with a shorter inter β-sheet distance in the assembled GY7 fibrils.^29^

### 3.2. Electrochemical impedance spectroscopy (EIS)

Electrochemical impedance spectroscopy (EIS) measurements to obtain information on the conductivity of the samples NY7, GY7 and SY7 at different temperatures, in the interval 20–160 °C, in steps of 20 °C, were carried out in the frequency range between 0.1 Hz to 10 MHz applying a voltage of 100 mV.

To evaluate the stability of the fibers at high temperatures, the fibers were heated to 110 °C for 10 minutes, and their amyloid nature was assessed after the samples were cooled to room temperature using Th-T dye (Fig. SI3,† panel A). In all samples, the emission signal from Th-T was comparable to that of fresh fibers. Additionally, the morphological changes were examined through TEM images. While some lateral disassembly of the fibers was observed, along with the presence of small amorphous aggregates in the TEM images, the fibers remained stable after heating (Fig. SI3,† panel B).

**Fig. 3:**
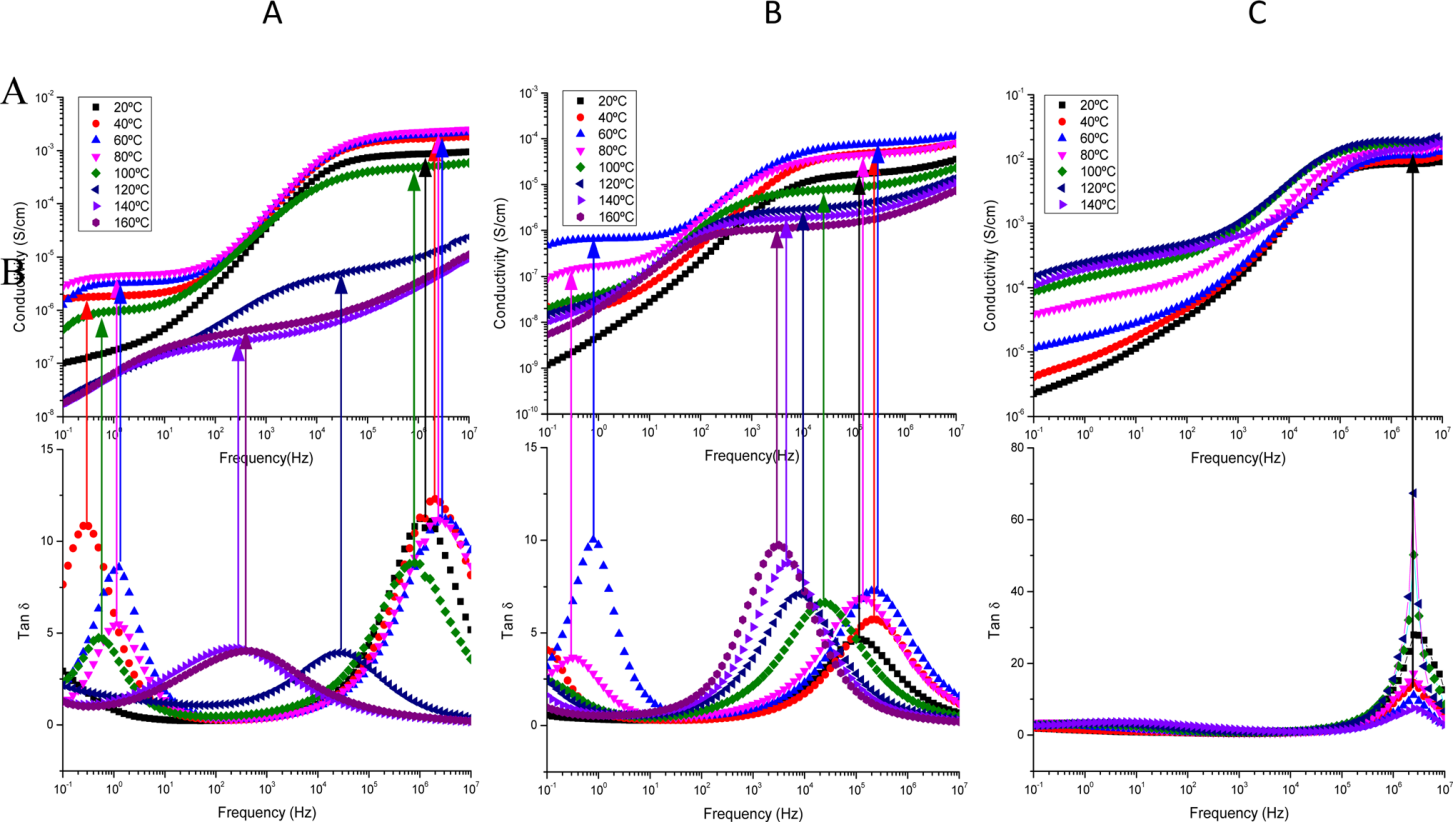
Conductivity (up) and tan *δ* (bottom) *versus* frequency in the interval of temperatures from 20 to 160 °C, in steps of 20 °C, for the samples GY7 (panel A), NY7 (panel B) and SY7 (panel C), measured in wet conditions. The arrows show the correspondence between the peaks of tan *δ* with the real part of the conductivity. Notice that at the frequency peak in tan *δ* we determine the dc-conductivity of the peptide at each temperature.

From dielectric spectroscopy measurements, the real part of the conductivity was analyzed in terms of the corresponding Bode diagrams, where variations of the conductivity with the frequency for samples GY7 (panel A), NY7 (panel B) and SY7 (panel C), are represented in Fig. 3. In this plot, the double logarithmic plot of the real part of conductivity in S cm^−1^*versus* frequency in (Hz) in the range of analyzed temperatures from 20 °C to 160 °C is shown. In this figure the arrows show the correspondence between the peaks of loss tangent (tan *δ*) with the real part of the conductivity indicating the value of the frequency at which the conductivity of the peptides has been determined. A close inspection of Fig. 3 also shows that the real part of the conductivity of the samples has a different behavior depending on the temperature. Notice that in the region of low temperatures (between 20 °C to 100 °C), the conductivity of the sample GY7 presents a plateau at high frequencies. We observed that conductivity increased as frequency increases, reaching a plateau at a given frequency (*σ*′ is constant with the frequency), whereas the out of phase angle *ϕ* = tan^−1^(*Z*′′/*Z*′) reached a maximum (or generally tends to zero). Knowing that Z* = *Z*′ + *jZ*′′ is the complex impedance, while *Z*′ and *Z*′′ are the real and imaginary parts of the impedance, respectively. When lim |Z*| tends to a constant value and *ϕ* = 0, the ionic resistance of the samples, *R*_0_, can be obtained, then from *R*_0_ = |Z*| at tan^−1^(*Z*′′/*Z*′) = 0, and then the dc-conductivity, *σ*_dc_ is the constant value obtained from the plateau of the Bode diagram shown in Fig. 3, which value can be given as1
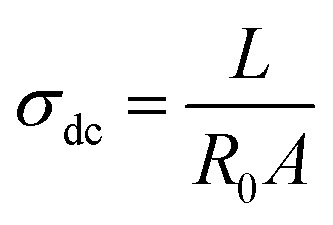
where the dc-conductivity *σ*_dc_, (S cm^−1^), is related with the impedance of the sample (*R*_0_ (Ω) = |Z*|^2^/*Z*′), the thickness of the protein *L* (cm) and the contact area of the sample sandwiched between electrodes *A* (cm^2^) by the eqn (1).

In some temperatures we observed two constant conductivity values of *σ* in a width range of frequencies in both GY7 and NY7 samples at high and moderate frequencies, such as is shown in Fig. 3 (panels A and B), while in case of SY7 the plateau of the conductivity is given at higher temperatures, such we can see in panel C of Fig. 3, although a slight tendency to a plateau at low frequencies seems to be appreciated also in the case of this sample. This indicates that the impedance comprises two resistive contributions, with the higher frequency value representing the direct current (dc) conductivity of the peptide. In Fig. 3, the top panels display the conductivity as a function of frequency for the GY7, NY7and SY7samples over a temperature range of 20 to 160 °C, with increments of 20 °C. In the bottom panels, the loss tangent is depicted for the GY7, NY7 and SY7 peptides, respectively, although in the SY7 the temperature range was from 20 to 140 °C.

The conductivity value for each peptide, measured in wet condition, can be determined from the intercept on the OY-axis, which corresponds to the intersection of the extrapolated frequency-independent plateau line. The conductivity plots at various temperatures (ranging from 20 °C to 80 °C) exhibit plateaus at both higher and lower frequencies. This result suggested the presence of two conductivities associated with different ion mobility and diffusivity in the respective peptides. Notice that both plateaus are associated with the two maxima observed in the plot of tan *δ*.

A close inspection of Fig. 3 shows that loss tan *δ* presents two maxima in the interval of temperatures compress between 20 °C and 100 °C, disappearing when the temperature increased until 160 °C. This behavior indicated the existence of two conductivities which should be associated with different mobility's and diffusivities of the ions in the different peptides. Such conductivities are obtained at the frequency where the loss tangent reach a maximum. On the other hand, at temperatures up to 100 °C, at frequencies lower than 10^2^ Hz the conductivity of the peptides showed a cut-off frequency where it started increasing with the frequency until to reach a new plateau in the high frequencies' region, which conductivity is higher than 500 times. This behavior is typical of samples whose behavior is not that of a pure conductor. This can be explained as a Debye relaxation due to the macroscopic polarization of the charges as a consequence of the electric field applied. This relaxation is characterized by a relaxation time, which is depending on the temperature and chemical structure. This performance can be due to the reorientation motion of dipoles and more likely to the motion of the localized charges, which are dominates over the dc-conductivity.^25,30–32^

On the other hand, in Fig. SI4,† it can be observed that the phase angle exhibits two maxima at the same temperatures where the conductivity curves displayed plateaus. One of the maximums is observed at high frequencies, while the other maximum is observed at low frequencies, indicating the existence of two distinct conductivities in the NY7 peptide. Similar observations were found for the GY7 and SY7 peptides. Additionally, the GY7 and SY7 samples revealed a plateau in the high frequency region, showing that its conductivities are approximately 1000 times higher than the conductivity observed at low frequencies, while for the NY7 sample was around 500 times higher, with a similar behavior. In all samples, the imaginary part of the impedance becomes zero or tends to zero, its mean that conductivity measured at high temperature represent the dc-conductivity of the samples. This behavior is the typical demeanor of an ionic solid electrolyte. It is worth to note that this behavior disappeared for both samples at high temperatures. However, the peptide SY7 exhibited higher conductivity values, with conductivity values reaching approximately 0.012 S cm^−1^ at temperatures below 100 °C such as we can see in panel C of Fig. 3.

Using the non-covalent interaction integral method and classical molecular dynamics, we have shown that the most favourable architectures for the amyloid assembly are those in which tyrosine residues are exposed to the solvent and polar residues are packed at the interface, forming a parallel polar zipper on either side of the extended β-filament.^29^ It is likely that the residue composition at the core may influence the conductivity of the different fibers.

**Fig. 4:**
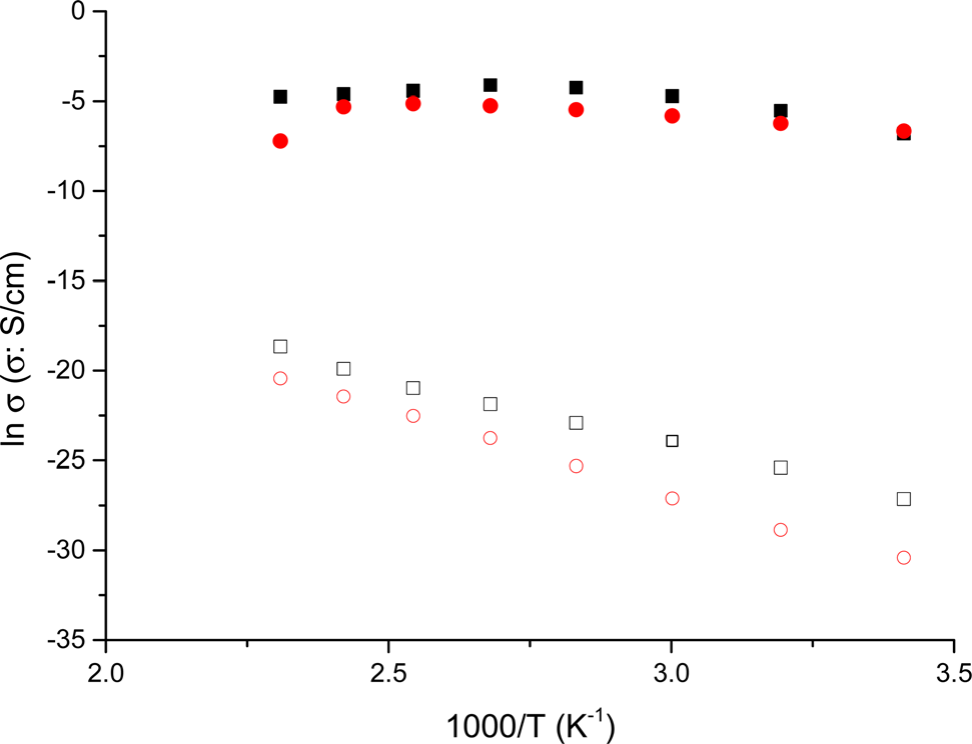
Arrhenius behavior of the conductivity for the Tyr – monomeric and Tyr – fibers under dry and wet conditions: (●) Tyr -monomer-wet, (■) Tyr -fib-wet, (○) Tyr -monomer-dry and (□) Tyr -fib-dry, respectively.

To assess the influence of neighboring amino acids such as Asn, Ser, and Gly, and their proximity to tyrosine on proton transport in peptides, we investigated the behavior of tyrosine monomeric and nanofibers in terms of conductivity. To achieve this, we examined the changes in conductivity with respect to temperature for the nanofibers, in both dry and wet conditions. This analysis allowed us to differentiate how these peptides behave when incorporated into the nanofibers. In Fig. 4 we plot the Arrhenius behavior of the conductivity for the Tyr-monomeric and Tyr fibers under dry and wet conditions.

A detailed analysis of Fig. 4 revealed the conductivity trends with temperature for Tyr monomeric and embedded into fibers under wet and dry conditions. This observation is particularly intriguing as it provides insights into the reproducibility of conductivity measurements for both Tyr monomeric and fibers. That is to say, we observe the same tendency for the Tyr than fibers in both conditions wet and dry, following a similar trend in both cases, although several orders of magnitude smaller in the case of dry than wet measurements. Interestingly, the variation of conductivity with temperature is nearly identical for both wet Tyr monomers and wet Tyr fibers, but slightly less in the case of Tyr monomers than fibers. On the other hand, when comparing dry Tyr monomers with Tyr nanofibers, the conductivity increased with temperature in both cases following a similar trend. However, the conductivity measured in dry conditions was higher, around one order of magnitude in the case of Tyr fibers than Tyr monomeric, suggesting that the alignment of the fibers enhances the transport pathways, thereby favoring conductivity. It is worth noting that although the experimental procedure in dry conditions aimed to remove water from the peptide by subjecting it to cycles from 20 to 100 °C prior to each measurement, there is still a possibility of water retention in the peptide matrix. This can be attributed to water molecules associated with the anions present in the peptide matrix. Taking into account the compared results observed between dry and wet conditions in Tyr monomers than fibers we have proceeded in the peptides to work under wet conditions.

**Fig. 5:**
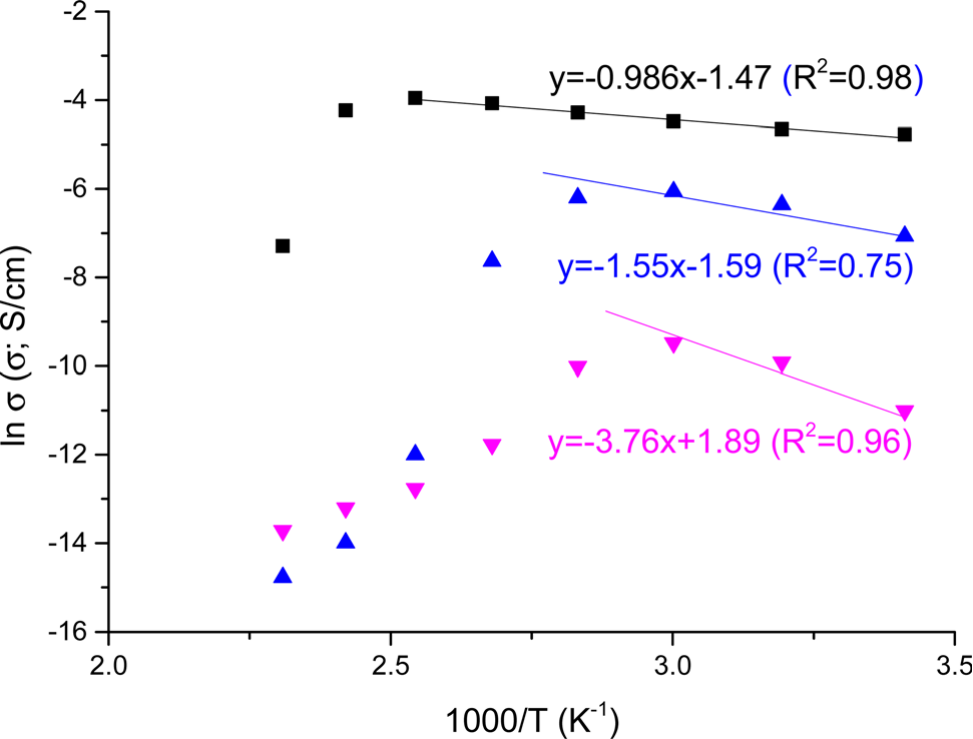
Conductivity variation with the reciprocal of temperature measured in wet conditions for the fibers SY7 (■), GY7 (▲) and NY7 fibers (▼), respectively. The lines are plotted to calculate the activation energy associated to the conductivity from the slope of the fits.

In Fig. 5 we illustrated the variation of conductivity with the reciprocal of temperature for NY7, GY7 and SY7-fibers in wet conditions, respectively. From this figure, it is evidenced that the through-plane conductivities of GY7-fibers-wet and SY7 fibers-wet increased with temperature within the range between 20 and 80 °C for GY7, and until 120 °C for SY7. However, for SY7 fibers-wet, there was a subsequent decrease in conductivity from 120 to 160 °C, whereas for GY7 fibers-wet, this decrease was much pronounced and started around 80 °C.

In contrast, the conductivity of NY7 fibers exhibited a sharp decrease from 60 °C onwards. This observation suggested that the conductivity of the fibers is strongly influenced by the presence of protons within them, with SY7 and GY7 demonstrating higher conductivity compared to NY7.

As general trend, NY7 fibers displayed lower conductivity values, around 0.04 ± 0.01 mS cm^−1^, meanwhile in GY7 and SY7 fibers the conductivities were, 1.7 ± 0.2 mS cm^−1^ and 9.1 ± 1.2 mS cm^−1^, respectively at 40 °C. On the other hand, at 80 °C the conductivities increased and the values observed were 13.8 ± 1.5 mS cm^−1^ for SY7, 2.0 ± 0.3 mS cm^−1^ for GY7 and 0.05 ± 0.01 mS cm^−1^ for NY7 fibers. Overall, the peptides showed conductivities following the trend *σ*(NY7) < *σ*(GY7) < *σ*(SY7), in all the range of temperatures studied; reaching maximum values of 19 mS cm^−1^ for the SY7 fiber, and 16 mS cm^−1^ for the GY7 fiber at 120 °C, under wet conditions procedure. The activation energy associated to the conductivity variation with the reciprocal of temperature showed in Fig. 5 followed the trend *E*_act_ (SY7) = 8.2 ± 0.6 kJ mol^−1^ < *E*_act_ (GY7) < 13 ± 5 kJ mol ^−1^< *E*_act_ (NY7) = 31 ± 7 kJ mol^−1^. Notice that these value were determinate from 20 to 120 °C for the peptide SY7, from 20 °C to 80 °C in case of GY7 and from 20 °C to 60 °C for NY7.

Under dry conditions, the dc-conductivity values were lower in comparison with wet conditions. In this work we have found the values 1.8 × 10^−5^, 1.7 × 10^−10^ and 3 × 10^−13^ S cm^−1^ at 40 °C for the SY7, GY7 and NY7 fibers at 40 °C, respectively. While at 80 °C, these values were 1.3 × 10^−7^, 2 × 10^−8^ and 1.3 × 10^−11^ S cm^−1^ for SY7, GY7 and NY7 fibers, respectively. The conductivity of SY7 was approximately one order of magnitude higher than that of GY7 and around 1000 times higher than NY7 at 80 °C, and even higher at lower temperatures (20 °C, 40 °C, and 60 °C) under dry conditions. The activation energy associated to the conductivity values measured following dry conditions has been obtained from the Arrhenius plot showed in Fig. SI5,† where the conductivity values were obtained following the same procedure than wet conditions. That is, from the Bode diagram where the real part of the conductivity are plotted *versus* frequency in the range of temperatures compress between 20 °C and 140 °C, for the samples SY7, GY7 and NY7, respectively. The activation energy of peptides under dry conditions follow the trend: *E*_act_ (SY7) = 26.5 ± 0.9 kJ mol^−1^ < *E*_act_ (GY7) = 59.1 ± 1.2 kJ mol^−1^ < *E*_act_ (NY7) = 77.5 ± 1.8 kJ mol^−1^. All the fits had a correlation coefficient of *r*^2^ = 0.99 in the same rage of temperatures analyzed (from 20 °C to 140 °C). Prior research has shown that the inclusion of a single acidic or basic amino acid into the side chain of a heptameric self-assembling peptide derived from the Ab peptide significantly amplifies proton conduction within the resulting fibers, increasing it by two orders of magnitude.^33^ Since the composition of the prion-inspired fibers lacks acidic or basic dissociation constants in their lateral chains, and the tyrosine (Tyr) content is consistent among the three fiber types, the disparities in energy values observed between these fibers may be ascribed to several factors. These factors encompass variances in the peptides' hydrophilicity, subtle distinctions in the distances between hydrogen bonds within the amyloid structure, the greater length of SY7 fibers relative to GY7 and NY7, and the potential variability in water retention capacity among the different fiber types.

In wet conditions, the presence of water molecules elevates the charge carrier density due to [H]^+^ ions associated with the water molecules, resulting in enhanced conductivity. The impact of protons on SY7 and GY7 fibers regarding conductivity can be explained in terms of changes in polarity and hygroscopicity related to the cation. Furthermore, for hydrated peptides, a substantial water ad layer envelops the peptide assemblies, establishing a network of hydrogen bonds that facilitates proton conduction through two distinct mechanisms. In the first mechanism, known as the Grotthuss mechanism, protons are transported through a thermally activated process. This mechanism is prevalent in hydrogen-bonded systems like water, where H^+^ ions follow the Grotthuss mechanism, leading to increased mobility. In this mechanism, H^+^ ions move swiftly as they transfer along a network of hydrogen bonds, essentially forming a proton wire *via* tunneling or hopping.^34^

Simultaneously, the presence of entropic barriers linked to the quantity of crosslinking agent (in our case, water) and the temperature, which regulates ionic transport within the peptide, may enable a vehicular-type mechanism. Both of these mechanisms are crucial for the overall charge conduction and have been observed in our experiments. Consequently, the density of proton/proton holes originating from the peptide side chains is higher.^35–37^ These processes are likely those found in polymer electrolytes, exemplified by polyvinyl alcohol nanofiber-reinforced Nafion® membranes, which exhibit a conductivity of approximately 22 mS cm^−1^.^11^

To put this into perspective, consider the proton conductivity of the Ampullae of Lorenzini (AoL) jelly, which exhibits an impressively high value at room temperature, measuring around 2 ± 1 mS cm^−1^.^38^ This conductivity is only 18 times lower than the current state-of-the-art proton-conducting polymer Nafion-115, as measured at 60 °C,^11^ and around 8 times lower than sPEEK-30% PVB nanofiber (where PVB stands for polyvinyl butyrate),^12^ and 6 times lower than our peptide SY7 studied in this work, which displayed a conductivity below 100 °C of 12 mS cm^−1^.

When comparing conductivity values to those reported in the literature, it becomes evident that the field of biomaterials has achieved conductivities, typically ranging from 10^−3^ S cm^−1^ to 10^−6^ S cm^−1^. In a recent study, Pena-Francesch and colleagues, explored the influence of tandem repetition on bulk proton conductivity within a family of highly stretchable and self-healing proteins inspired by squid ring teeth.^39^ Their findings demonstrated some of the highest reported conductivity values among biological materials, reaching up to 3.5 mS cm^−1^. Furthermore, Migliaccio and colleagues recently reported a noteworthy breakthrough: the electrical conductivity of eumelanin, thanks to a straightforward thermal annealing process in a vacuum. This induced structural reorganization of the molecular constituents, resulting in an astounding value of 318 S cm^−1^, marking the highest conductivity observed to date.^40^ As for the most remarkable conductivity reported for an amyloid microcrystal containing a single Tyr residue, it stood at 3.5 ± 0.96 μS cm^−1^, demonstrating intrinsic electronic conductivity facilitated by micrometer-long hole hopping *via* tyrosine's.^37^

### 3.3. Mobility, diffusivity, and free charge density

The mobility and free charge density can be obtained from the measurements of loss tangent.^13–25^ Considering that the anion and cation have the same mobility, *μ*, and neglecting the ion–ion interaction, the dc-conductivity is given by the general ionic conductivity equation

2 *σ* = *nqμ*

where *μ* is the mobility, *q* the charge of a monovalent ion and *n* is the equilibrium number density of free charges, *i.e.* the total proton concentration in the peptide. Eqn (2) assumes that the cation transfer number is equal to one, being only applicable in the case of anions that are practically immobile. This approximation is plausible taking into account that Tyr anions derived from the peptide are covalently linked to the polymeric chains and assuming a reduced mobility for the anion as a consequence of its larger size in comparison with that of the cations (H^+^ or H_3_O^+^). Therefore, the mobility is restricted to the anions until the system reach the equilibrium, where the concentration of cations (H^+^ or H_3_O^+^) will be dependent of the temperature, as described Einstein relationship as:3
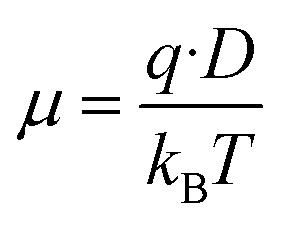
where *D* is the diffusion coefficient, *T* the absolute temperature, and *k*_B_ is Boltzmann's constant.

In our study, ion–ion interactions were neglected, and the electroneutrality at the equilibrium condition was maintained for the fibers. Then, the ionic concentrations are *n*_1_(eq) = *n*_2_(eq) = *n*_p_, defining *n*_p_ as the equilibrium number density of free positive charges (*i.e.*, the total cation concentration in the peptide). For such suppositions and considering the Nernst–Einstein relation, the mobile charge concentration, *n*_p_, can be written as^18,21^4
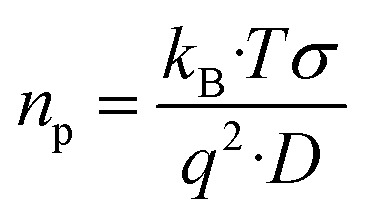
where *k*_B_ is the Boltzmann constant (*i.e.*, 1.38 × 10^−23^ J K^−1^), *T* the absolute temperature, *F* the Faraday's constant (96 485.33 C mol^−1^), *q* is the charge of a monovalent cation (1.6 × 10^−19^ C), *σ* the dc-conductivity of the peptides (in S m^−1^) and *D* the diffusion coefficient of the cations (in m^2^ s^−1^). The eqn (4) permits us to determine the density of cations (H^+^ or H_3_O^+^) (in m^−3^) from the values of the ionic conductivity measured in (S m^−1^), from the frequency domain where the loss tangent reach a maximum in the dielectric spectra of Bode diagram, determining the diffusion coefficient according to the equation.^41–43^5
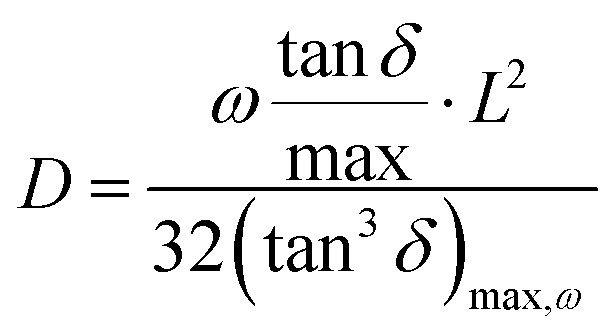
where, *L* is the thickness of the peptide, 
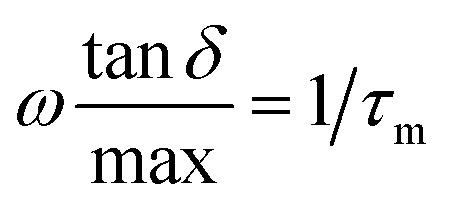
, being *τ*_m_ the reciprocal of the relaxation time corresponding to the frequency where tan *δ* shows a maximum. This relaxation time is associated to the ionic conduction mechanism in the peptides, and 
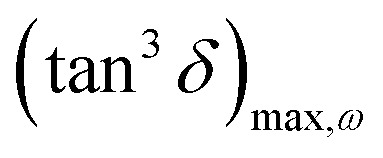
 the value of the loss tangent at the peak. From Fig. 3 we can observe at some temperatures two peaks for all peptides. This means the possible existence of two possible conduction mechanisms within the temperature range studied. One of them at high frequencies (short relaxation times) and could be related to the formation of dipoles and the movement of the charge carriers, protons and H_3_O^+^ in our case, while the other at low frequencies closely related to the mobility of the charges may be associated to the impurities and hydrogen bonding formation in the peptides.

In Table 2, we have gathered the values obtained for the diffusivities (*D* in m^2^ s^−1^) obtained using eqn (5) for the GY7, NY7 and SY7 fibers, respectively, as a function of the temperature, considering the observed peaks at high and low frequencies in loss tangent of Fig. 3. In the same table, we have tabulated the values of the charge carrier's density (*n*) of the same peptides at the same temperatures in m^−3^. Based on our results, it is evidenced that diffusivity can be influenced by the presence of two distinct mechanisms of conduction Grotthuss and vehicular, where both are influenced possibly to the existence of impurities which diffusivities are lower than the protonic diffusion but make the transport of protons difficult. Furthermore, we observed a correlation between diffusivity and both the density of the charge carriers and the temperature.

**Table 2:** Values of diffusion coefficients and mobile charge concentration obtained at different temperature, from the peaks observed in loss tangent for the peptides NY7, GY7 and SY7, respectively

Sample	*T*(°C)	*D*_1_(m^2^ s^−1^)	*D*_2_(m^2^ s^−1^)	*n*_1_(m^−3^)	*n*_2_(m^−3^)
NY7	20	4.8 × 10^−6^	—	5.4 × 10^19^	—
	40	6.9 × 10^−6^	2.9 × 10^−12^	1.2 × 10^20^	2.9 × 10^26^
	60	7.2 × 10^−6^	4.1 × 10^−12^	1.9 × 10^20^	3.4 × 10^26^
	80	1.8 × 10^−6^	12.2 × 10^−12^	4.8 × 10^20^	7.0 × 10^25^
	100	3.9 × 10^−7^	—	3.9 × 10^20^	—
	120	1.0 × 10^−7^	—	6.0 × 10^20^	—
	140	3.4 × 10^−8^	—	1.2 × 10^21^	—
GY7	20	7.0 × 10^−6^	3.9 × 10^−12^	1.9 × 10^21^	3.4 × 10^27^
	40	1.1 × 10^−5^	9.9 × 10^−12^	2.8 × 10^21^	3.0 × 10^27^
	60	2.2 × 10^−5^	2.0 × 10^−11^	1.7 × 10^21^	1.9 × 10^27^
	80	1.8 × 10^−5^	4.6 × 10^−11^	2.5 × 10^21^	9.8 × 10^26^
	100	1.1 × 10^−5^	6.5 × 10^−11^	8.5 × 10^20^	1.5 × 10^26^
	120	4.1 × 10^−6^	—	3.2 × 10^19^	—
	140	3.4 × 10^−8^	—	2.4 × 10^20^	—
SY7	20	3.2 × 10^−7^	—	8.8 × 10^23^	—
	40	7.8 × 10^−7^	—	1.0 × 10^23^	—
	60	1.4 × 10^−6^	—	5.7 × 10^22^	—
	80	2.1 × 10^−6^	5.6 × 10^−11^	3.4 × 10^22^	1.9 × 10^26^
	100	2.2 × 10^−6^	7.0 × 10^−11^	2.2 × 10^25^	6.0 × 10^25^
	120	1.1 × 10^−7^	1.2 × 10^−10^	2.5 × 10^25^	6.1 × 10^25^

**Fig. 6:**
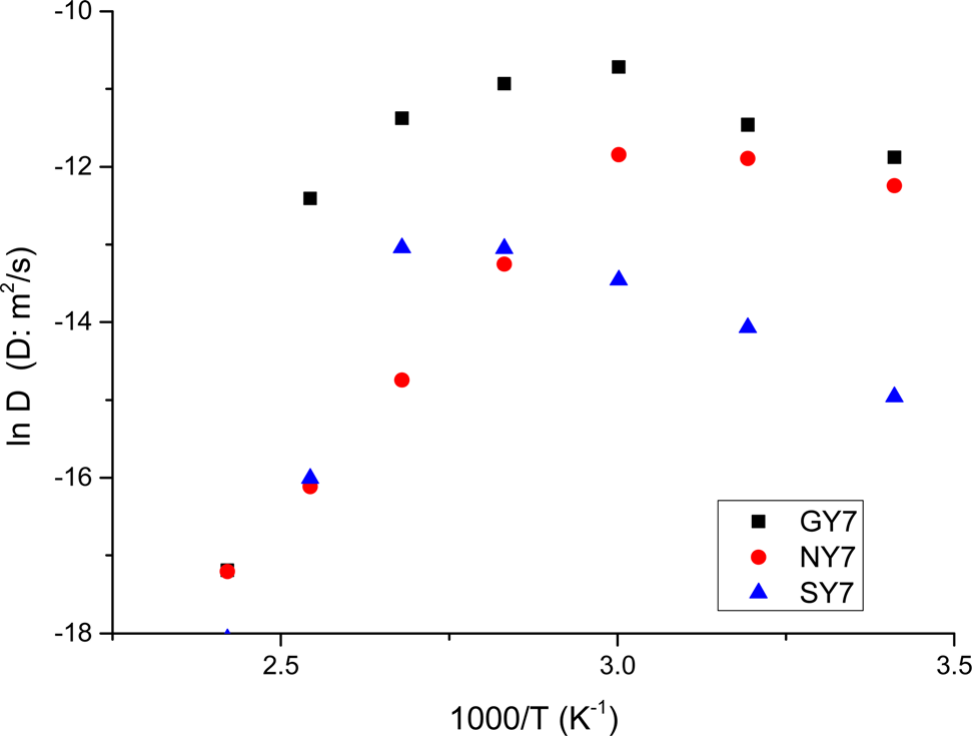
Temperature dependence of diffusion coefficient for the peptides. NY7 (●), GY7 (■) and SY7(▲).

From the values gathered in Table 2 and plotted in Fig. 6, it is apparent that the diffusion coefficient correlated with increasing temperature in NY7 fibers for temperatures under of 50 °C, while in GY7 fibers for temperatures compress between 20 °C and 60 °C, and in case of SY7 between 20 °C and 80 °C. Below these temperatures the diffusivity decrease when the temperature increase. However, it is noteworthy that the diffusivity observed in the SY7 peptide is lower, compared to GY7 and NY7 presumably due to its enlarged length. This observation can be attributed to two factors: firstly, the higher conductivity values observed in SY7 compared to GY7 and NY7, possibly due to the existence of minus impurities, and secondly, to the value of relation 
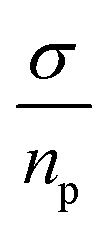
 observed of cations present in the peptide SY7 compared with GY7 and NY7, which in turn is dependent on temperature.

Resuming a close inspection of diffusion coefficient values revealed that all peptides exhibit a good ion diffusivity (*i.e.* mobility). The comparison between the fibers showed a decrease in the fiber linearity together with a decreasing of water content leads to a reduction of diffusivity around one order of magnitude depending of the temperature. Moreover, the peptide SY7 retain more quantity of water than QY7 and NY7, and therefore their conductivities remain elevated at temperatures above 100 °C, because water has a local vehicle/carrier function for excess H^+^ and H_3_O^+^. From these results, we suppose that SY7 fibers maintain a higher water content in its structure compared to the others fibers. This can be due to the presence of favorable polar zipper architectures, which create a strong network of hydrogen bonds and promote the packing of Tyr at surface.

## 4. Discussion

Recognizing the urgent necessity to establish model systems that can unravel the intricacies of charge transport within proteins, our research was ignited by the amino acid composition of PrDs. This inspiration guided the design of four different short polar self-assembling peptides, leading to the formation of fibrillar structures with the ability to facilitate proton conduction.

Our study reveals the significant potential of Tyr-containing peptides as redox-active scaffolds to develop biocompatible catalysts. In particular, our synthetic NY7, GY7, and SY7 peptides show good dissociative molecular exchange properties, in particular the peptide SY7, which shows proper conductivity and diffusivity. Our results illustrate how the structure-compactness of Tyr-enriched fibers directly impacts the conductivity with peptide NY7 showing conductivity values approximately two orders of magnitude lower than peptides GY7 and SY7 at 40 °C. The conductivities observed were 13.8 ± 1.5 mS cm^−1^, 2.0 ± 0.3 mS cm^−1^ and 0.05 ± 0.01 mS cm^−1^, for the peptides SY7, GY7 and NY7, respectively at 80 °C. The values of the proton conductivity found for the peptides SY7 and GY7 are higher than the value obtained for Ampullae of Lorenzini jelly (AoL jelly) which value at room temperature was (2 ± 1) mS cm^−1^.^38^ Therefore, our peptides shown conductivity values which can be satisfactory to be potentially exploited for a variety of applications, including transparent conductive coatings, electronic circuits, capacitors, and sensors.

The proton conduction activity of the short self-assembled peptides might find application in the creation of proton exchange membranes for fuel cells, enabling the efficient movement of protons between anode and cathode, and ion-selective membranes. Our findings may hold implications for the next generation of biocompatibles proton conducting materials and protonic devices because their good conductivity can be used to create high-performance proton-conducting membranes for energy storage devices, like as batteries and supercapacitors.

Overall, the versatility and biocompatibility of Tyr-rich self-assemblies offer great potential for diverse nanotechnological and biomedical applications.

## 5. Conclusion

In conclusion, our study successfully designed short polar self-assembling peptides inspired by PrDs, resulting in fibrillar structures capable of efficient proton conduction. The significant potential of Tyr-containing peptides as redox-active scaffolds for biocompatible catalysts was revealed, with peptide SY7 demonstrating significant conductivity and diffusivity. The conductivity values observed in the peptides SY7, GY7, and NY7 hold promise for various applications, including conductive coatings, biosensors, and tissue engineering scaffolds.

Moreover, the proton conduction activity of these short self-assembled peptides opens up opportunities in the development of bioelectrical interfaces for ion-selective membranes.

Altogether, the versatility and sustainability of Tyr-rich self-assemblies not only provides insightful understandings of the structure–property relationship but also offer great potential as versatile, sustainable and smart material for diverse nanotechnological and biomedical applications. The outcomes detailed in this study make a meaningful contribution to the expanding domain of biomaterials research and underscore the promising outlook of these peptide-based structures in shaping forthcoming innovative technologies.

## Conflicts of interest

The authors declare that they have no known competing financial interests or personal relationships that could have appeared to influence the work reported in this paper.

## Supplementary Material

NA-006-D4NA00303A-s001
