# Supplementary material for: Harnessing prion-inspired amyloid self-assembly for sustainable and biocompatible proton conductivity
Source: Nanoscale Adv. 2024 Apr 17;6(10):2669–81. doi: 10.1039/d4na00303a (PMC11093263; doi:10.1039/d4na00303a)
Supplement: NA-006-D4NA00303A-s001 [file NA-006-D4NA00303A-s001.pdf]

Electronic Supplementary Material (ESI) for Nanoscale Advances.
This journal is © The Royal Society of Chemistry 2024

Supplementary Information

Harnessing Prion-Inspired Amyloid Self-Assembly for Sustainable and Biocompatible Proton Conductivity

Susanna Navarro[1*], Andreu Andrio[2], Marta Diaz-Caballero[1], Salvador Ventura[1], Vicente Compañ[3,*]

[1] Institut de Biotecnologia i Biomedicina and Departament de bioquímica I Biología Molecular, Universitat Autónoma de Barcelona, 08193 Bellaterra, Barcelona, Spain.

[2] Dpto. Física. Universitat Jaume I, Avda. Sos, Baynat s/n, Castellon 12071, Spain.

[3] Escuela Técnica Superior de Ingenieros Industriales, Departamento de Termodinámica Aplicada, Universitat Politècnica de València, Camino de Vera s/n, 46020 Valencia, Spain.

* Correspondence: V. Compañ. E-mail: vicommo@ter.upv.es; and Susanna Navarro. E-mail: Susanna.Navarro.Cantero@uab.es

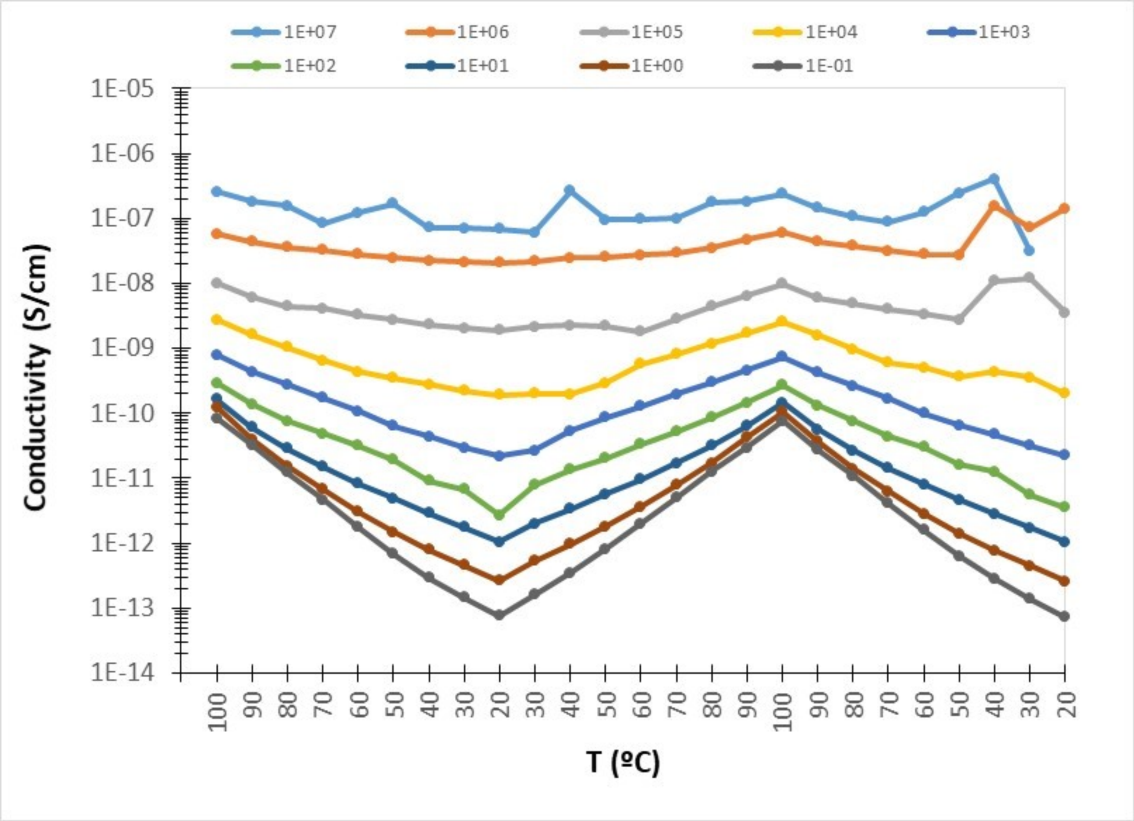

Figure SI1. Reproducibility of the measurements after applying two cycles of heating and cooling in each cycle from 20 to 100 ºC. In this plot the results for the NY7 peptide are represented. The colour of the lines in the figure is the frequency for the which the conductivity has been measured at the given temperature.

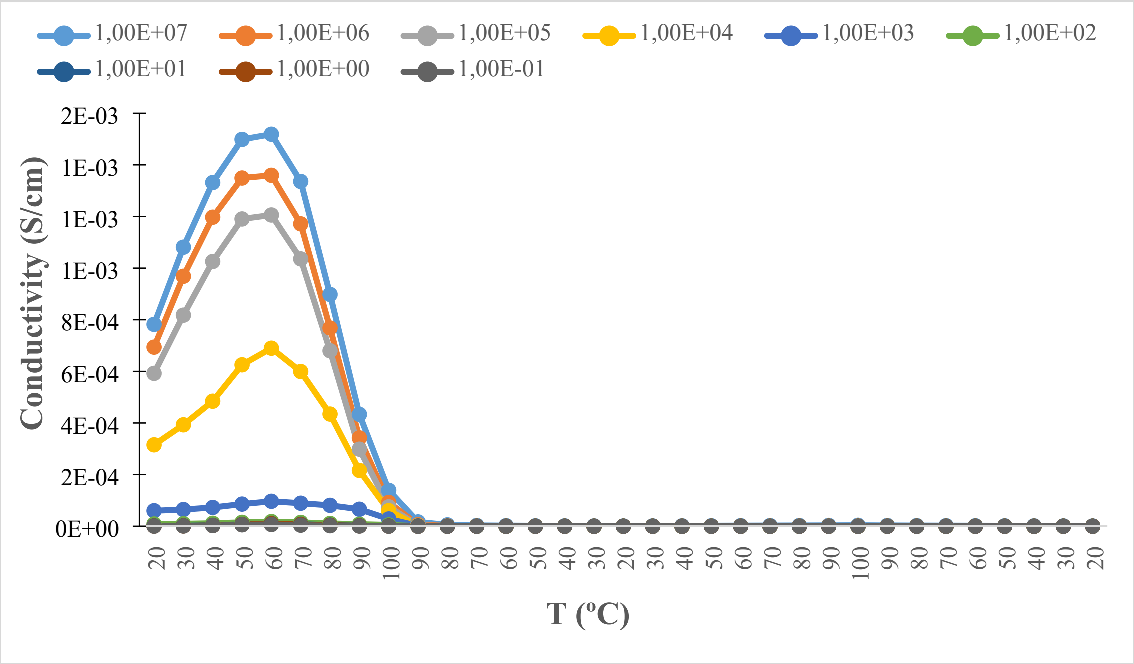

Figure SI2. Reproducibility of the measurements after applying two cycles of heating and cooling in each cycle from 20 to 100 ºC. In this plot the results for the GY7 peptide are represented. The colour of the lines in the figure is the frequency for the which the conductivity has been measured at the given temperature.

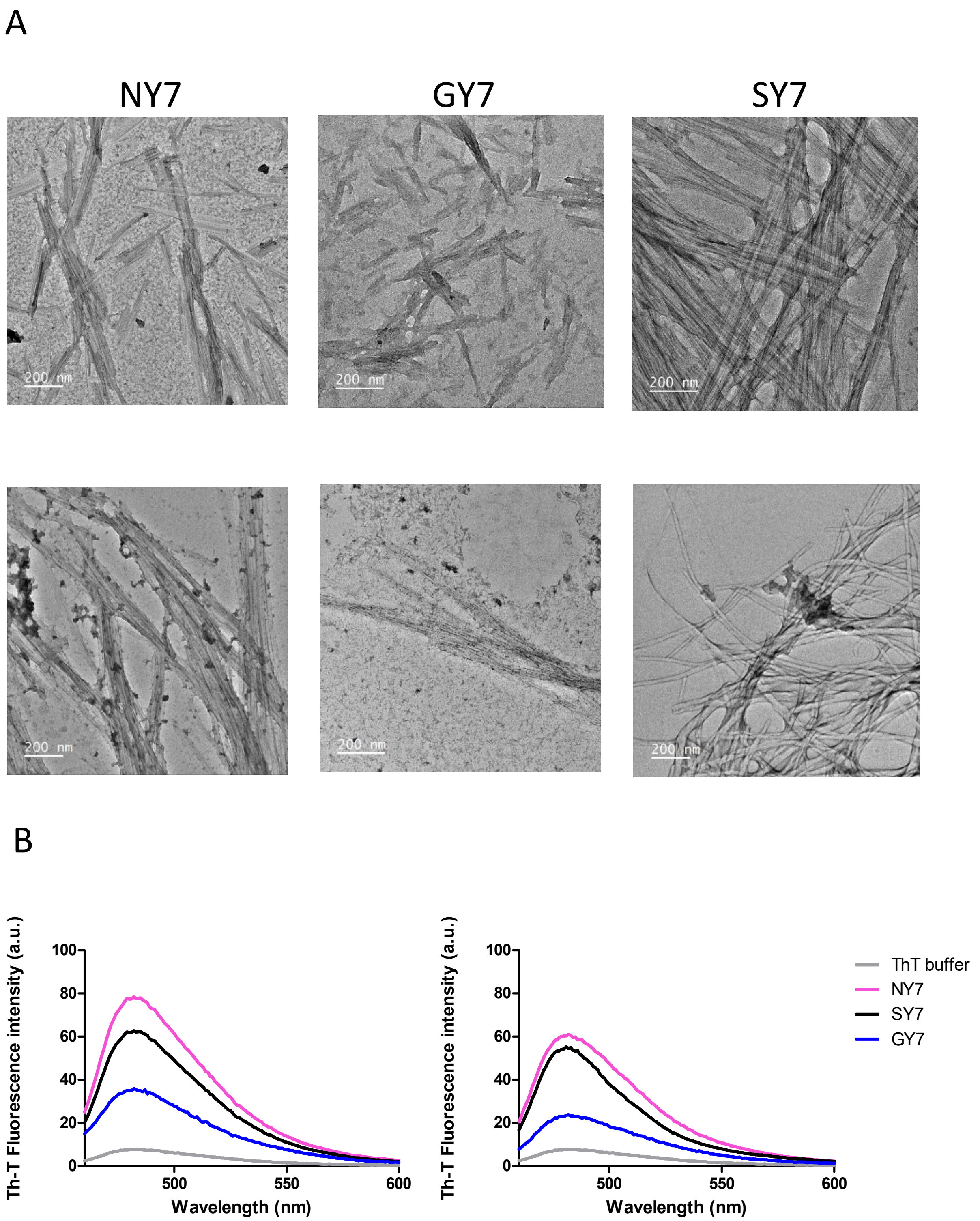

Figure SI3. Characterization of the amyloid properties of Tyr-enriched heptapeptides fibers (NY7, SY7, and GY7) before and after heating. A) The morphology of samples was assessed by TEM. Representative micrographs of fresh fibers (upper row) and fibers incubated at 110ªC (bottom row). Scale bars correspond to 200 nm. B) Fluorescence emission spectra of Th-T recorded with fresh (left) and heated fibers at 110ºC (right).

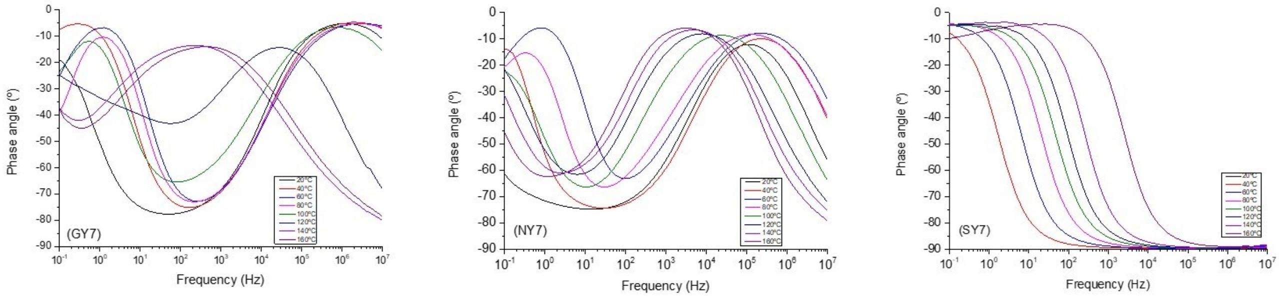

Figure SI4. Phase angle *versus* frequency for the peptides GY7, NY7 and SY7, respectively at all temperatures studied.

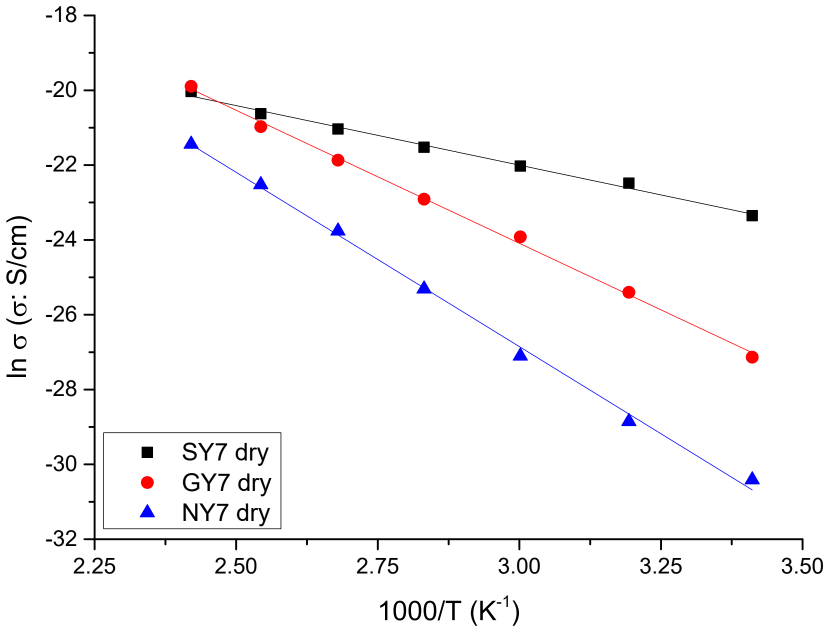

Figure SI5. Conductivity variation versus the reciprocal of temperature measured under dry conditions for the fibers SY7 (O), GY7 (℘) and NY7 (□), respectively in the range of temperatures compress between 20ºC and 140ºC.
